# The effect of spatial position and age within an egg-clutch on embryonic development and key metabolic enzymes in two clownfish species, *Amphiprion ocellaris* and *Amphiprion frenatus*

**DOI:** 10.1371/journal.pone.0226600

**Published:** 2020-01-08

**Authors:** Andreas Kunzmann, Valeska C. Diemel

**Affiliations:** 1 Leibniz Centre for Tropical Marine Research, Fahrenheitsstraße, Bremen, Germany; 2 University of Bremen, Bibliothekstraße, Bremen, Germany; Tokat Gaziosmanpasa University, TURKEY

## Abstract

Since the size of newly hatched larval fish is directly related to egg size, small differences in initial egg size can be critical to survival and further development of offspring. Underlying processes causing size variation in fish offspring are still not entirely understood. In this study we investigated whether the spatial position of an individual egg within a clutch affects size variation in two benthic spawning coral reef fishes, the clownfishes *Amphiprion ocellaris* and *A*. *frenatus*. To evaluate the effects of within-clutch position on embryonic development, egg growth metrics and protein content were analysed on day 2, 5 and 8 after deposition (adp). Additionally the activities of the key metabolic enzymes citrate synthase (CS) and lactate dehydrogenase (LDH) were investigated to evaluate the physiological status of the embryos. Central eggs of *A*. *frenatus* were significantly longer and heavier than peripheral eggs only on day 2 and 5 adp (2.07 mg, 2.59 mm vs. 1.84 mg, 2.49 mm). No significant differences were observed in *A*. *ocellaris* between eggs originating from a central or peripheral (5 mm from edge) position (1.33 mg, 2.26 mm vs. 1.15 mg, 2,18 mm). Diameter of the eyes did not differ between the two fish species nor between different positions, for any age group. The protein content of eggs (7.5% of wet weight) was independent of age, position and species. Enzymatic activity increased from 2 adp until peak activity was observed for both enzymes on day 8 adp, independent from position. The range of CS- and LDH-activity was 0.3–13.0 and 0.2–71.7 U g^-1^ wet weight, respectively. Significant differences in enzymatic activity were observed between age groups in both species, which in connection with significantly larger eggs of *A*. *frenatus* at day 2 and 5 adp could hint at a better O_2_ supply of central eggs. Potential implications for captive breeding are given.

## Introduction

More than 2 billion people worldwide are believed to keep marine aquaria and global ornamental fish trade has increased from US$ 50 to US$250 million over the past two decades [1, 2, 3]. About 24 billion fish are collected and transported every year, mainly from coral reefs in Southeast-Asia to be sold on markets in Europe and North-America [1]. The most traded species are coral reef fishes, whose wild populations are heavily under pressure. This includes several species of the benthic spawning clownfish, above all *Amphiprion ocellaris* (Aoc) and *Amphiprion frenatus* (Afr). To protect wild populations of clownfish it is necessary to breed them in captivity, but despite the high economic interest, little research on the physiology of clownfish, especially in early life stages, is done. Breeding in captivity still leads to a certain degree of mortality of larvae during their first days of life [3, own observations].

In marine fish the body size at hatching plays an important role, because bigger larvae tend to have higher chances of survival and faster development than smaller larvae [4]. There are three main factors [5] driving variation in the development of embryos: 1. the maternal resources (genetic differences, quality of the egg, arrangement of the clutch), 2. the local environment (gas and heat exchange, water quality parameters) and 3. in benthic spawning fish the parental care, which can even modify the local environment [5, 6]. In 2006 Green et al. [5] investigated for the first time the effect of within-clutch position on the development of eggs of a clownfish clutch (*A*. *melanopus*). They discovered that eggs from the periphery were >2% smaller in mean body length and 4–6% smaller in mean volume than eggs from the centre of the clutch and postulated a better oxygen supply in the centre. The eggs from the periphery also had a 33% lower rate of oxygen consumption per egg (2.89 x10^-5^ compared to 4.31 x10−^5^ μmol O_2_ egg ^-1^ s^-1^) than the embryos from the clutch interior. These previous results suggest a role of metabolism / oxygen supply in driving the effect of position on development.

The activities of key metabolic enzymes in organs and tissues are important indicators, which can give information on the physiologic and metabolic state of an animal [7], particularly in combination with oxygen consumption rates. To analyse the aerobic/anaerobic metabolism, many studies use the oxidative enzyme citrate synthase (CS) and the glycolytic enzyme lactate dehydrogenase (LDH) [8, 9, 10, 11, 12]. While enzymatic measurements have never been done before in embryos of clownfish and the impact of egg position within the clutch on enzyme activities is unknown, we hypothesize that in eggs, which develop faster or lead to larger larvae, oxygen consumption is higher and also the enzyme activity would be elevated [7, 13] and that egg position matters [5]. In addition, the activities of CS and LDH will reveal, whether oxidative or glycolytic pathways are preferred [9].

Therefore, this study investigated the link between age and egg position (centre/periphery) within a clutch with egg standard length (SL), egg diameter, egg wet weight, eye diameter and protein content. In addition, this study provides the first data about the metabolism during embryonic development in the two species of clownfish, Aoc and Afr.

## Materials and methods

Seven adult pairs of Aoc (7.3–8.1 cm total length) and three pairs of Afr (11.3–11.8 cm total length) were kept in a 4000l seawater tank. These animals were kept in the MAREE research aquaria systems of ZMT for many years and were originally bought from an ornamental trader. Ten 200 L Tanks were provided with earthenware tiles for hiding and spawning. Water parameters were controlled daily (Salinity 32 PSU; Temperature 28° C; pH: 8.1; O_2_ concentration > 95%). Fish were fed with frozen food (*Artemia*, *Mysis*, *10*:*1*) twice a day at 9 am and 4 pm. In nature clownfish attach 300 to 700 eggs in single-layer circular clutches to hard structures near to their host anemone. The parents tend the clutch until the well-developed embryos hatch after 7–8 days after deposition (adp).

**Fig 1:**
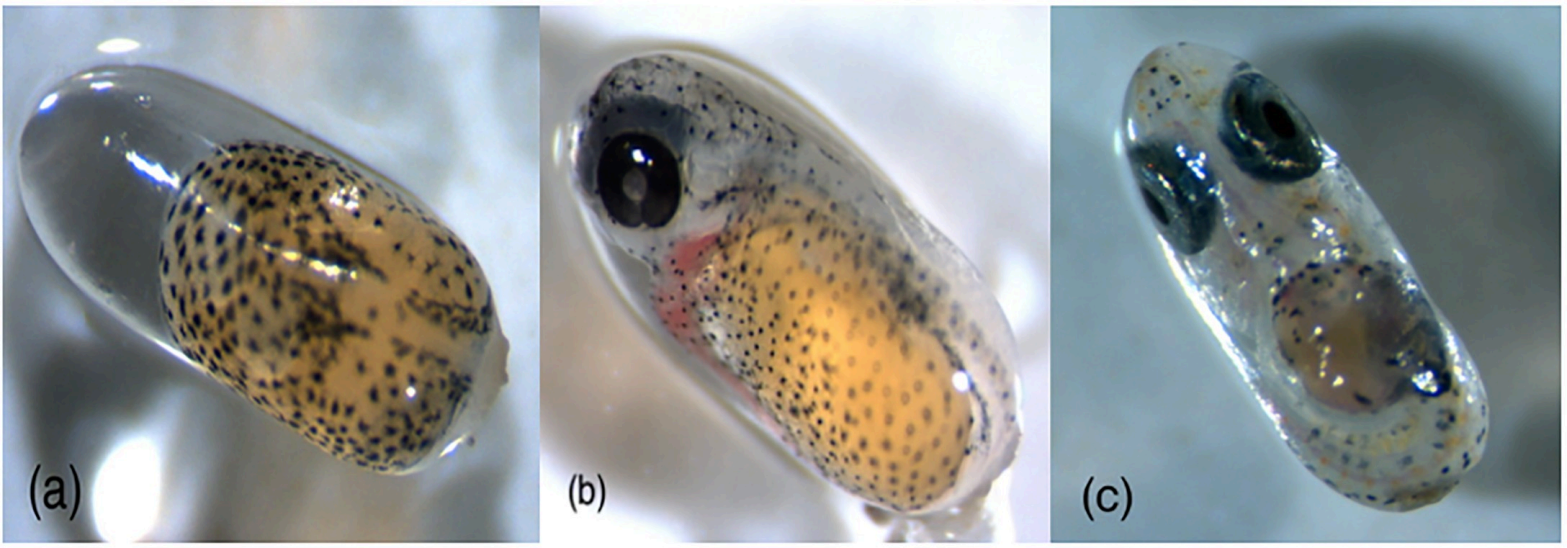
Eggs of *Amphiprion ocellaris* on (a) day 2, (b) day 5 and (c) day 8 of embryogenesis.

To examine size variation of embryos within a clutch, eggs were sampled at three developmental stages, day 2, 5 and 8 adp (Fig 1). Each of the 33 clutches was divided into two positions: periphery (5 mm from the edge) and centre (the remaining embryos). Eggs were removed from the slabs with a scalpel, rinsed with distilled water, transferred into a petridish and stored on ice. The wet weight (ww) of 10 eggs per position was measured first, then the measurements of 10 additional eggs per position were taken. Data are given in mean value +/- standard error (n is indicated in the graphs). Eggs were photographed under a dissecting binocular with camera attachment. Images were captured and SL, egg diameter and eye diameter were measured using *Adobe Photoshop*. Although eye size is not expected to change in response to environmental conditions [14], it provides a standard for comparison with changes in the other variables. During the measurements eggs were stored on ice to avoid enzymatic degradation.

Measurements of enzymatic activities were done after Sidell et al. for citrate synthase CS, Hickey and Clements for pyrovate kinase PK and Lushchak et al. for lactated dehyrodgenase LDH [15, 16, 17]. Protein content determination was done after Bradford [18]. For these measurements 45 eggs per species in total were used, data are given in mean value +/- standard error (n is indicated in the graphs).

For statistical analysis Linear Mixed Models LMM and Generalized Linear Mixed Models GLMM and R Version 3.5.1 were used. The effect of age and position in clutches on the different growth parameters and enzymatic activities was tested for both species separately. Differences between peripheral and central regions of the clutches, among age groups, and their interactions were assessed using mixed modeling approaches. Normality of the residuals was visually assessed using normal QQ-plots. Scatterplots of residuals vs fitted values were used to assess heteroscedasticity. R Version 3.5.1 (R Core Team, 2018) with the extension packages nlme, lme4, and lmerTest were employed for statistical analysis.

Linear Mixed Models (LMM) were fitted when residuals were homoscedastic and deviated not, or only marginally, from normality. If necessary, the VarIdent function of the nlme package was used to correct for heteroscedasticity. If model assumptions could not by satisfied by the use of a LMM, Generalized Linear Mixed Models (GLMM) with a Gamma distribution and either a log- or canonical-link function were fit to the data instead (see Table 1 for details). Models were simplified until only significant variables remained. As eggs in the same clutch and clutches from the same breeding pair are likely to be more similar, the clutch ID, nested in the breeding pair, was incorporated as a random variable to achieve independence. Age was used as a factor variable as it was the goal to assess differences among ages rather than relationships over time.

**Table 1:** Tested variables and applied models.

Tested Variable	Model: *Amphiprion ocellaris*	Model: *Amphiprion frenatus*
Wet-weight	GLMM (Gamma distribution with log-link function)	LMM with correction for heteroscedasticity
Standard Length	LMM	LMM
Egg Volume	LMM	LMM
Egg Diameter	LMM	LMM with correction for heteroscedasticity
Eye Diameter	LMM	LMM with correction for heteroscedasticity
CS Activity	GLMM (Gamma distribution with canonical-link function)	GLMM (Gamma distribution with canonical-link function)
LDH activity	GLMM (Gamma distribution with canonical-link function)	GLMM (Gamma distribution with canonical-link function)
Protein Content	GLMM (Gamma distribution with log-link function)	LMM

The tested variables are given in Table 1 and the results of the final, reduced models are summarized in Tables 2 and 3.

**Table 2:** Results of final, reduced models for *Amphiprion ocellaris*.

Response	Predictor(s)	Model	Estimate	p-value
**Wet-weight**	Position	GLMM (Gamma, log-link)	Intercept: 0.186 ± 0.106 SE Periphery: -0.131 ± 0.007 SE	0.079 <0.001
**Standard Length**	Position	LMM	Intercept: 2.235 ± 0.0893 SE Periphery: -0.072 ± 0.007 SE	<0.001 <0.001
**CS Activity**	Age	LMM (Gamma, canonical-link)	Intercept: 1.226 ± 0.252 SE Age 5: -1.106 ± 0.070 SE Age 8: -1.340 ± 0.078 SE	<0.001 <0.001 <0.001
**LDH Activity**	Age	GLMM (Gamma, canonical-link)	Intercept: 0.566 ± 0.042 SE Age 5: -0.462 ± 0.045 SE Age 8: -0.515 ± 0.044 SE	<0.001 <0.001 <0.001

**Table 3:** Results of final, reduced models for *Amphiprion frenatus*.

Response	Predictor(s)	Model	Estimate	p-value
**Wet-weight**	Position Age	LMM VarIdent: Age	Intercept: 2.367 ± 0.125 SE Age 5: -0.028 ± 0.176 SE Age 8: -0.324 ± 0.179 SE Periphery: -0.393 ± 0.030 SE Age 5 *: 0.183 ± 0.032 SE Age 8 *: 0.083 ± 0.054 SE	<0.001 0.875 0.010 <0.001 <0.001 0.122
**Standard Length**	Position	LMM	Intercept: 2.730 ± 0.059 SE Periphery: -0.120 ± 0.008 SE	<0.001 <0.001
**CS Activity**	Age	GLMM (Gamma, log-link)	Intercept: -0.395 ± 0.178 SE Age 5: 0.594 ± 0.068 SE Age 8: 1.823 ± 0.083 SE	0.026 <0.001 <0.001
**LDH Activity**	Age Position	GLMM (Gamma, log-link)	Intercept: 0.522 ± 0.326 SE Age 5: 1.236 ± 0.132 SE Age 8: 2.949 ± 0.157 SE Periphery: -0.344 ± 0.076 SE Age 5 *: 0.520 ± 0.107 SE Age 8 *: 0.343 ± 0.107 SE	0.110 <0.001 <0.001 <0.001 <0.001 0.001

Ethics: The experiments followed the rules of the ethics committee of the University of Bremen, we followed the recommendations of the Bremen Senate’s office for animal ethics (Senator für Gesundheit, Permit from 04.04.2013).

## Results

**Fig 2:**
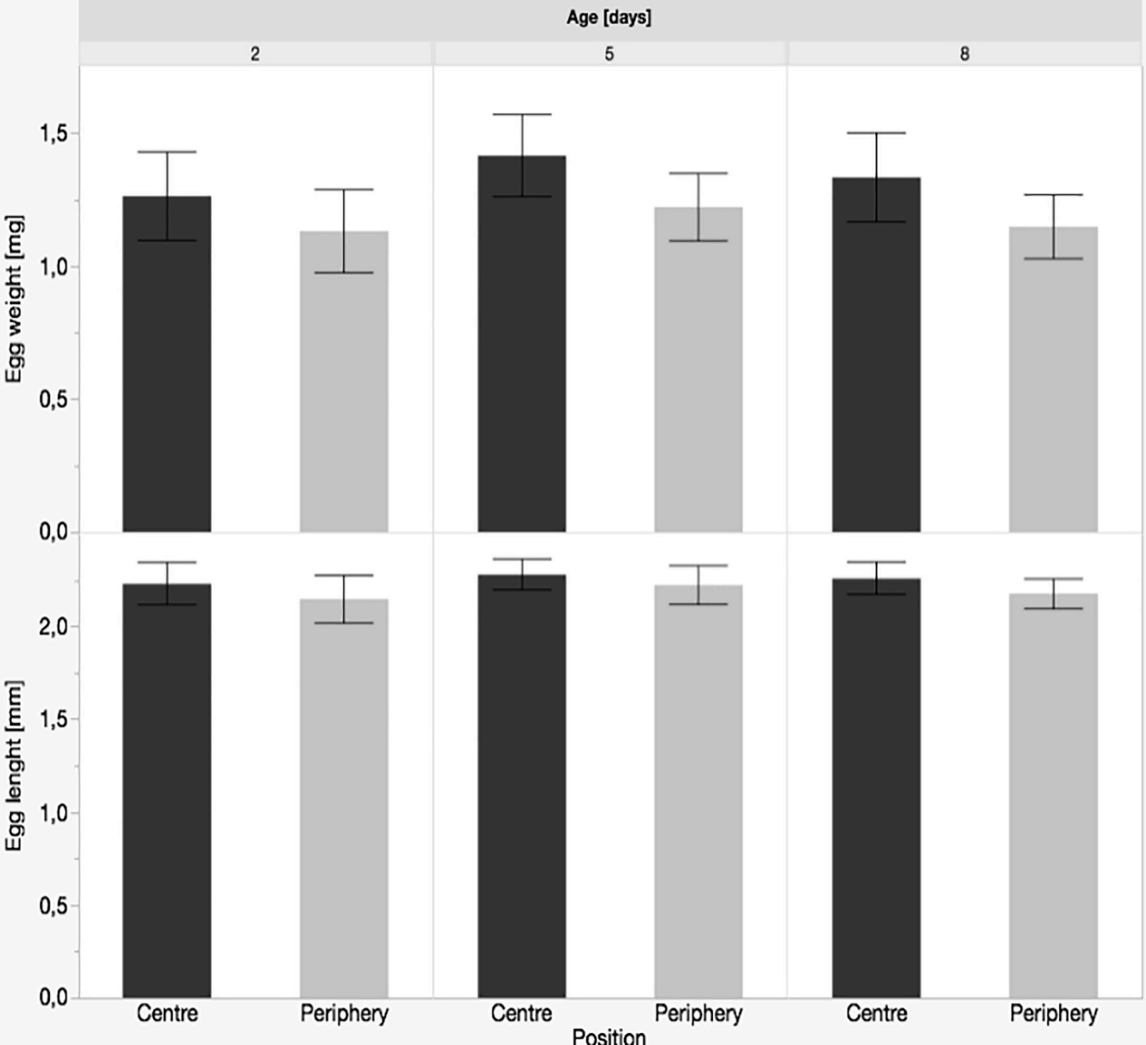
Egg weight and length of a) *Amphiprion ocellaris* (10 eggs each from 6 clutches) and b) *A*.*frenatus* (10 eggs each from 5 clutches) by age and position. No significant differences were found in *A*. *ocellaris*, while * significant differences between centre and periphery in *A*. *frenatus* are indicated with different letters (p < 0.001).

We analysed eggs of 18 Aoc clutches and 15 Afr clutches. In eggs of Aoc no significant size differences between eggs from the centre and the periphery were found. At day eight of development the following min-max ranges were found: egg ww 1.1–1.4 mg, SL 2.1–2.3 mm, egg diameter 0.90–0.94 mm and eye diameter 0–0.5 mm. In contrast to Aoc, eggs of Afr displayed a significant difference in ww only on day 2 adp (heavier in the centre, p = 0.001) and in SL after 5 days adp (larger in the centre, p = 0.001). At day eight of development ww was 1.8–2.3 mg, SL was 2.5–2.8 mm, egg diameter was 0.96–1.1 mm and eye diameter 0–0.5 mm. In both species we found a strong tendency towards bigger and heavier eggs in the clutch centre, but few significant differences (Fig 2).

**Fig 3:**
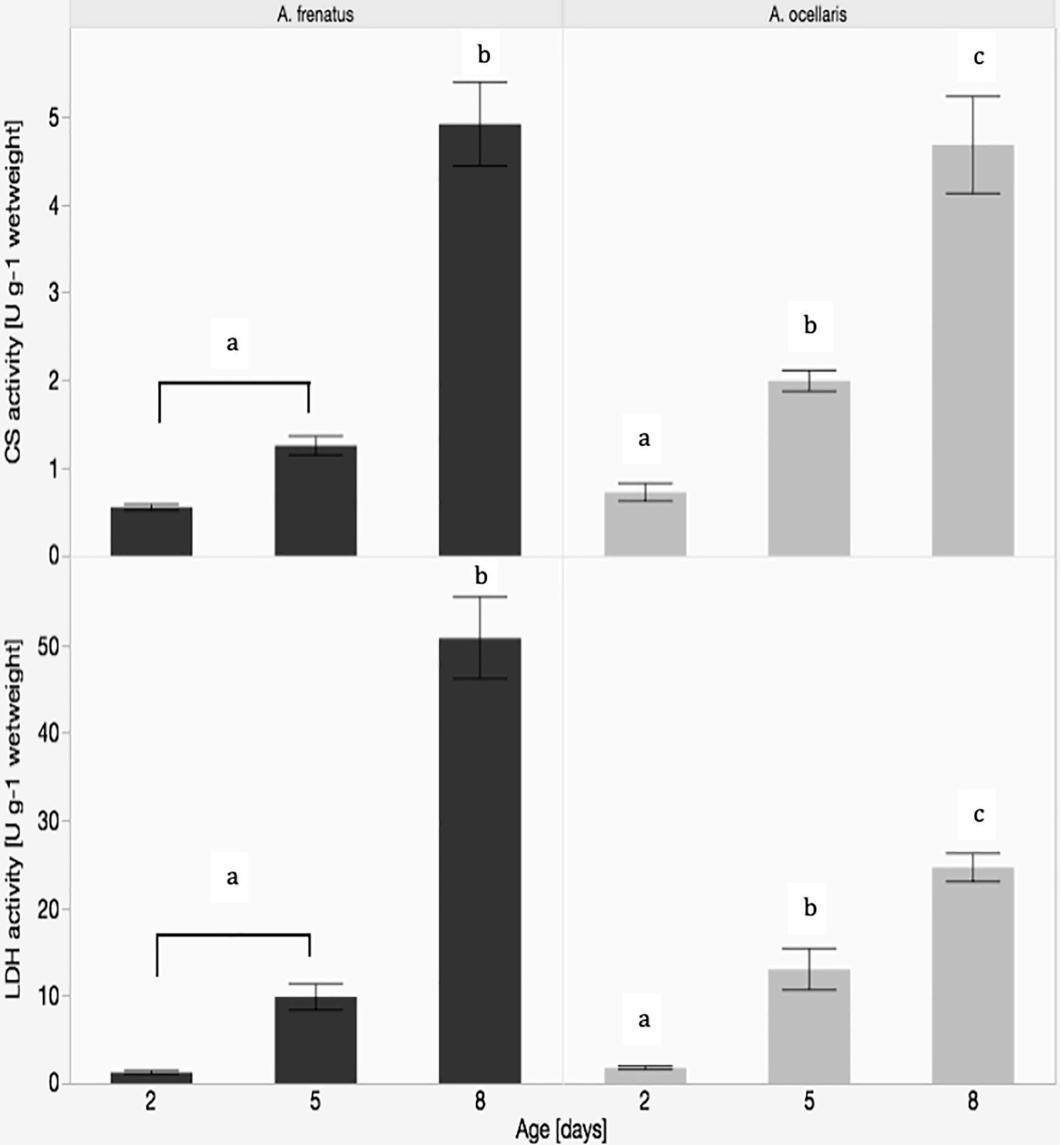
Enzyme activity of CS and LDH for *Amphiprion ocellaris* (n = 12) and *A*.*frenatus* (n = 10) by age. Different letters indicate significant differences (p < 0.001). In the case of *A*. *ocellaris* all activities are significantly different from each other, while in the case of *A*. *frenatus* there is no significance between days 2 and 5, but between days 5 and 8.

Citrate synthase (CS) activities of Aoc were increasing from day 2, over day 5 to day 8 adp of development (significant differences see Fig 3). Since we could not find significant differences between eggs from the centre and the periphery we combined the data. On day 2 adp eggs had a CS activity of 0.727 ± 0.101 U g^-1^ ww. On day 5 adp CS activity was significantly higher (p < 0.001) with 1.991 ± 0.552 U g^-1^ ww. Significantly highest CS activities were measured on day 8 adp (p < 0.001) with 4.679 ± 0.552 U g^-1^ ww. Lactate dehydrogenase (LDH) activities of Aoc were also significantly different on all three days of development. On day 2 adp eggs had a LDH activity of 1.776 ± 0.187 U g^-1^ ww. On day 5 adp LDH activity was significantly higher (p < 0.001) with 13.003 ± 1.35 U g^-1^ ww. Also here significantly highest LDH activities were measured on day 8 adp (p < 0.001) with 24.612 ± 1.613 U g^-1^ ww.

Since we could not find significant differences between eggs from the centre and the periphery in Afr, also here we combined the data. CS and LDH activities of Afr on day 2 adp were 0.550 ± 0.034 U g^-1^ ww and 1.245 ± 0.237 U g^-1^ ww, respectively. Only day 8 adp was significantly different (p < 0.001) with 6.096 ± 0.934 U g^-1^ ww 58.249 ± 6.637 U g^-1^ ww, respectively. The protein content of the eggs was independent from age and position. We measured a protein content of 6.3–8.0% of ww in Aoc and of 7.0–8.9% of ww in Afr. The mean protein content of the eggs was 7.4 ± 0.2% of ww.

When we compare both species, the ww, SL and egg diameter showed significant differences in almost all ages. Values are always higher in Afr than in Aoc. CS activities in Afr were only significantly higher on day 5 adp (p = 0.001), while LDH activities showed significant higher values on day 8 adp (p < 0.001). Activities of CS and LDH increased after day 5 adp stronger in Afr than in Aoc. In contrast, the protein content did not differ significantly between species.

## Discussion

In this study we investigated the links between age, spatial position within egg clutches of two clownfish species and growth as well as enzyme properties. This is the first report on the development of the key metabolic enzymes CS and LDH during embryogenesis in Aoc and Afr.

Ww, SL and egg diameter in Aoc and Afr showed only small differences during the embryonic development. These measured values match with the results of previous studies [19, 20, 21]. Aoc showed a tendency towards bigger and heavier eggs in the centre compared to eggs from the periphery, but differences were not significant. Afr also showed a tendency towards bigger and heavier eggs in the centre, with significant differences in ww on day 2 adp and in SL on day 5 adp. Most of the differences were probably not significant, because of the small number of samples. These differences in egg size could mean either that (1) females invest differently into eggs from the centre and periphery, because central eggs are better protected against predators, or (2) that the spatial pattern of the clutch favours the growth of central eggs. Green *et al*. [5] could show that the females of *Amphiprion melanopus* lay their eggs in zigzag patterns onto the substrate. These findings support that the assumption (1) is unlikely, because central eggs are laid interspersed with peripheral eggs. Size differences of embryos are more likely a result of the clutch configuration, but the mechanisms driving them are still unknown. One possibility would be an enhanced metabolism of the central embryos through an increased temperature due to a density effect in the clutch interior. Water temperature has a direct impact on the development of tropical fish at all live stages. 7 days old larvae of *A*. *melanopus* raised at 25°C took 25% more time to reach metamorphosis and were significantly smaller than larvae raised at 28°C [13]. In embryos of the tropical reef fish *Siganus randalli* the development of eyes began 3 hours earlier when eggs were kept at 30°C compared to the eggs kept at 27°C [22].

We found in both species that CS activity was lower (Aoc: 2.53 ± 0.37 U g^-1^ ww; Afr: 2.63 ± 0.55 U g^-1^ ww) than LDH activity (Aoc: 13.5 ± 1.94 U g^-1^ ww; Afr: 23.12 ± 5.15 U g^-1^ ww) during the whole embryogenesis. Our findings match with the results of previous studies. Jesben [23] showed that in white muscle tissue of bony fish the glycolytic enzymes are up to ten times more active than enzymes of the citrate cycle. They also found a much higher content of white muscle tissue compared to red muscle tissue in bony fish. While we were not able to distinguish between red and white muscle, it is assumed that because the oxygen transport in eggs and embryos relies on diffusion only, we measured higher values for the glycolytic enzyme activity (LDH) than for the oxidative enzyme activity (CS) [15, 17]. Highest enzyme activities were observed on day 8 adp, shortly before hatching. The increasing enzyme activities over time show the development of a yolk filled egg to a larva with fully developed organs and respiration system. The small enzyme activities on day 2 adp show the development of the embryo’s brain, which begins to develop very early in embryogenesis [18]. Enzyme activities increase strongly from day 5 to day 8 adp, due to the development of muscles, jaw and digestive system, which begins after day 5 adp [19, 20].

The protein content of eggs was independent from position, age and species. However, we could observe a trend to a slight decrease in protein content on day 5 and 8 adp. Mean values of combined data for both species show a protein content of 8.42 ± 0.36% ww on day 2 adp, 6.64 ± 0.32% ww on day 5 adp and 7.08 ± 0.33% ww on day 8 adp. The energy for the quick development of embryos comes from the yolk proteins inside the egg [24]. Yolk proteins are mainly and directly used to build up white muscle tissue and organs, and to a lower degree for cell respiration. Due to this there is only little loss of total protein content in the eggs, but mainly a protein turnover [24]. This process is also shown by our data, because protein content does not differ significantly between age groups, while the embryos perform an intense development. Because enzymes consist mainly of proteins, the increasing enzyme activities of CS and LDH show a changing protein composition within the embryos. With progressive embryogenesis the proteins in the yolk are used to build up enzymes (which again build up structural proteins). For a fast embryogenesis, like in clownfish, aerobic and anaerobic metabolism is needed to ensure a fast growth and development.

Eggs of Aoc and Afr differ in all measured characteristics, but eye diameter and protein content. Values for the SL, ww and egg diameter are always significantly higher in Afr than in Aoc. The size differences between the eggs are possibly due to the size differences of the adult fish, where females of Afr are bigger and heavier than females of Aoc [6].

In this study we could only show that central eggs of *A*. *frenatus* are heavier on day 2 adp, and larger on day 5 adp, while for day 8 it is not significant. For *A*. *ocellaris* only a trend towards larger eggs was visible. Size differences might be a result of nest configuration, enhanced by influences from the clutch micro-environment such as O_2_ supply. Large individuals from central locations may have a greater probability of survival, due to their embryonic micro-environment. Clutch position may be an important factor, determining survival in different developmental stages. In addition, we saw that glycolytic enzyme activities are higher than oxidative enzyme activities, at least during the first days. Enzymatic activities however increased significantly until day 8 adp. While our data are not sufficient to show this, it could be due to more effective oxygen supply, which might finally lead to higher metabolism, faster growing embryos and larger larvae [5, 9].

Implications for captive breeding could be that larger females (producing larger eggs) should be selected and the fanning activities or the parents should be supported by constant flow and aeration, as sufficient oxygen supply, particularly to the larger central eggs, could yield larger juveniles with better chances for survival. For future studies we suggest to analyse amino acid and fatty acid compositions of the eggs. Also, larval rearing studies should be performed, in order to evaluate the hatching and survival percentage of the smaller and bigger sized eggs, and particularly follow the fate of larger eggs.

## References

[1] Wabnitz C, Taylor M, Green E, Razak T. From Ocean to Aquarium. UNEP-WCMC, Cambridge, UK 2003; 64 pages.

[2] Rhyne AL, Tlusty MF, Szczebak JT, Holmberg RJ. Expanding our understanding of the trade in marine aquarium animals. Peer J 2018; 5:e2949; 10.7717/peerj.2949 28149703 PMC5274522

[3] Dhaneesh KV, Ajith Kumar TT, Divya SP, Kumaresan S, Balasubramanian T. Influence of prompt first feeding on growth and survival of Clownfish *Amphiprion percula* larvae. Emir. J. Food Agric. 2012; 24 (1): 92–97.

[4] Vigliola L, Meekan MG Size at hatching and planctonic growth determine post-settlement survivorship of a coral reef fish. Oecologia 2002; 131: 89–93. 10.1007/s00442-001-0866-4 28547516

[5] Green BS, Anthony KRN, McCormick MI. Position of egg within a clutch is linked to size and hatching in a demersal tropical fish. J exp Mar Biol Ecol. 2006; 329: 144–152.

[6] Swagat Gosh TT, Kumar A, Balasubramanian T. Determining the level of parental care relating fanning behaviour of five species of clownfish in captivity. Indian Journal of Geo-Marine Sciences 2012; 41(5): 430–441.

[7] Almeida-Val VMF, Gomes ARC Lopes NP. Metabolic and physiological adjustments to low oxygen and high temperature in fishes of the Amazon. The Physiology of Tropical Fishes. 2006; 21(10): 443–500.

[8] Cooper RU, Clough LM, Farwell MA, West TL. Hypoxia-induced metabolic and antioxidant enzymatic activities in the estuarine fish *Leiostomus xanthurus*. J exp Mar Biol Ecol. 2002; 279: 1–20.

[9] Hochachka PW, Somero GN, Schneider DE, Freed JM. The organization and control of metabolism in the crustacean gill. Comp. Biochem. Physiol. 1970; 33: 529–548.

[10] Lannig G, Eckerle LG, Serendero I, Sartoris F-J, Fischer T, Knust R, et al Temperature adaptation in eurythermal cod (*Gadus morhua*): a comparison of mitochondrial enzyme capacities in boreal and arctic populations. Mar. Biol. 2003; 142: 589–599.

[11] Kamyab E, Kühnhold H, Novais SC, Alves LMF, Indriana L, Kunzmann A, et al Effects of thermal stress on the immune and oxidative stress responses of juvenile sea cucumber *Holothuria scabra*. J. Comp. Physiol. B 2016; 10.1007/s00360-016-1015-z 27439718

[12] Kühnhold H, Kamyab E, Novais S, Indriana L, Kunzmann A, Slater M, et al Thermal stress effects on energy resource allocation and oxygen consumption rate in the juvenile sea cucumber, *Holothuria scabra* (Jaeger, 1833). Aquaculture 2016; 467:109–117. 10.1016/j.aquaculture.2016.03.018

[13] Green BS, Fisher R. Temperature influences swimming speed, growth and larval duration in coral reef fish larvae. J exp Mar Biol Ecol 2004; 299:115–132.

[14] Fuiman L, Poling L, Higgs D. Quantifying Developmental Progress for Comparative Studies of Larval Fishes. Copeia. 1998; 602–611.

[15] Sidell BD, Driedzic WR, Stowe DB, Johnston IA. Biochemical correlations of power development and metabolic fuel preferenda in fish hearts. Physiological Zoology 1987; 60(2): 221–232

[16] Hickey AJR, Clements KD. Key metabolic enzymes and muscle structure in triplefin fishes (Tripterygiidae): a phylogenetic comparison. Journal of comparative physiology 2003; 173(2): 113–23. 10.1007/s00360-002-0313-9 12624649

[17] Lushchak VI, Bagnyukova TV, Storey JM, Storey KB. Influence of exercise on the activity and the distribution between free and bound forms of glycolytic and associated enzymes in tissues of horse mackerel. Brazilian Journal of Medical and Biological Research 2001; 34(8): 1055–64. 10.1590/s0100-879x2001000800013 11471046

[18] Bradford MM. A rapid and sensitive method for the quantitation of microgram quantities of protein utilizing the principle of protein-dye binding. Anal Biochem. 1976; 72: 248–254. 10.1006/abio.1976.9999 942051

[19] Yasir I, Qin JG. Embryology and early ontogeny of an anemonefish *Amphiprion ocellaris*. J. Mar. Biol. Ass. U. K. 2007; 87: 1025–1033.

[20] Madhu R, Madhu K. Successful captive breeding and juvenile production of the tomato anemonefish, *Amphiprion frenatus*. Marine Fisheries Information Service, 2010; No. 205.

[21] Madhu R, Madhu K, Retheesh T. Life history pathways in false clownfish *Amphiprion ocellaris* Cuvier, 1830: A journey from egg to adult under captive condition. J. Mar. Biol. Ass. India. 2012; 54(1): 77–90.

[22] Collins LA, Nelson SG. Effects of temperature on oxygen-consumption, growth and development of embryos and yolk-sac larvae of *Siganus randalli* (Pisces, Siganidae). Marine Biology. 1993; 117: 195–204.

[23] Jebsen JW. Proteins in fish muscle. Fiskeridirektoratets kjemis tenise Forskningsinstitutt, Bergen.1962; 16 pages

[24] Smith RW, Ottema C. Growth, oxygen consumption, and protein and RNA synthesis rates in the yolk sac larvae of the African catfish (*Clarias gariepinus*). Comp. Biochem. Physiol. A. 2006; 143: 315–325. 10.1016/j.cbpa.2005.12.005 16426879

